# Suicide deaths and substance use in Spain between 2010 and 2022

**DOI:** 10.3389/fpsyt.2024.1435031

**Published:** 2024-09-30

**Authors:** Gerardo Flórez, Ashkan Espandian, Teresa Seoane-Pillado, Noelia Llorens, José Manuel Gerpe, Pilar Saiz

**Affiliations:** ^1^ Addictive Treatment Unit, Ourense University Hospital, Ourense, Spain; ^2^ Centre for Biomedical Research in the Mental Health Network (CIBERSAM), Oviedo, Spain; ^3^ Psychiatry Service, León Hospital, León, Spain; ^4^ Area of Preventive Medicine and Public Health, Department of Health Sciences, University of A Coruña - INIBIC, A Coruña, Spain; ^5^ Spanish Observatory of Drugs and Addictions, Government Delegation for the National Plan on Drugs, Spanish Ministry of Health, Madrid, Spain; ^6^ Demographic Surveys Section, Spanish Office for National Statistics, Orense, Spain; ^7^ Department of Psychiatry University of Oviedo, Oviedo, Spain; ^8^ Health Research Institute of the Principality of Asturias (ISPA), Oviedo, Spain; ^9^ University Institute of Neurosciences of the Principality of Asturias (INEUROPA), Oviedo, Spain; ^10^ Health Service of the Principality of Asturias (SESPA), Oviedo, Spain

**Keywords:** alcohol, completed suicide, illegal substances, joinpoint regression, preventive strategies

## Abstract

**Background:**

Suicide is a serious public health problem that affects our entire country. The aim of this research was to study the variation in completed suicide rates between 2010 and 2022 in Spain and their relationship with the consumption of addictive substances.

**Methods:**

Completed suicide data from the Spanish Statistical Office (INE) were analyzed with a joinpoint regression model to determine time trends. The relationship between the variation in completed suicide rates with sociodemographic variables, including depression rates, obtained from the Spanish Statistical Office and variables related to the consumption of substances obtained from the survey on alcohol and other drugs in Spain (EDADES) of the Government Delegation for the National Plan on Drugs (DGPNSD) was also studied using an exploratory analysis and also performing a Generalized Additive Model.

**Results:**

The joinpoint regression model did reveal a point of significant change in the period studied for Spain showing a trend of increased suicide death rates for the studied period. The following variables correlated positively with the variation in completed suicide rates in the Generalized Additive Model: alcohol use in the past 12 months, alcohol use in the last 30 days, daily alcohol use in the last 30 days, binge drinking in the last 12 months, binge drinking in the last month, positive Alcohol Use Disorder Test for risky alcohol use, benzodiazepine use in the last 12 months, benzodiazepine use in the last month, daily benzodiazepine use in the last month, use of illegal substances in the last 12 months, use of illegal substances in the last month, mean age and depression rates.

**Conclusion:**

Applying preventive strategies on the risky consumption of alcohol, benzodiazepines and illegal substances would help reduce the rates of completed suicide in Spain.

## Introduction

Suicide represents a serious public health problem and is one of the leading causes of death worldwide. Comorbidity with substance use significantly increases the vulnerability and risk of ideation, attempts, and completed suicide (1). This situation leads to the urgent need to improve current identification, prevention, and treatment strategies.

According to the latest data provided by the World Health Organization (WHO), the number of deaths due to suicide reaches nearly 700,000 people per year. The global rate is estimated at 9.4 suicides per 100,000 inhabitants, with a higher percentage in the male sex. In Spain, according to data from the Spanish Statistical Office (INE) (2), the highest number of deaths by suicide in history was reached in 2022, surpassing those of 2021 by 5.6 percent. Unfortunately, the upward trend continues, and this has been the main external cause of death since 2008, surpassing deaths due to traffic accidents (3) and representing the second leading cause of death in the young adult population (15-29 years) worldwide (4). At the level of the autonomous regions, the high suicide rates in Asturias followed by Galicia stand out, like previous years.

Risk estimation is a complex process due to the very nature of suicidal behavior, as well as to the difficulties encountered in its investigation. The importance of knowledge, identification and recording of sociodemographic variables as well as risk and protective factors for optimal clinical intervention of patients at risk of suicide stands out. The existence of mental disorders represents the main risk factor, with depressive disorder and alcohol consumption disorders being the most prevalent in relation to suicidal behavior (5, 6).

Suicide is the leading cause of death in people with substance use disorders (7). It is estimated that the risk of death by suicide compared to the general population increases 10-fold for people with alcohol use disorders and up to 22-fold for those addicted to other substances (8, 9). Alcohol plays a relevant role not only as a risk factor, but also as a precipitating factor, due to the disinhibition and executive dysfunction produced by alcohol intoxication (10). Neurobiological and psychological factors justify the association of alcohol consumption with suicide and an increased risk of death by suicide of 94% in the presence of alcohol consumption (11). It is estimated that the increased risk is so significant that the overall alcohol consumption of a population is associated with the prevalence of suicidal risk, which is why alcohol abuse is one of the most prevalent mental disorders in suicide deaths, second only to depressive disorders (11–13). The increased risk is highlighted by its association with mental illnesses such as depression, schizophrenia, and personality disorders (14). Intoxication by alcohol consumption increases the lethality of the method used, increasing the probability of a lethal outcome. One third of suicides test positive for alcohol, 63% of which show blood levels equivalent to intoxication (13).

Opiates are another of the most frequently detected intoxicants in completed suicides, at similar levels to alcohol and far removed from other intoxicants. They increase the risk of suicide 14 times more than in the general population and their association with alcohol, benzodiazepines or other mental illnesses makes them lethal (13, 15).

Of the prevention strategies currently implemented, only a minority enjoy any degree of evidence sufficient to ensure that they can reduce the incidence of suicide (16, 17). Work continues to identify the mainstay of psychological intervention (18). The latest prevention strategies currently being implemented -mainly in developed countries- focus on dividing the population into risk groups. Consequently, different psychological intervention strategies are applied to different risk groups (19). In Spain, priority is being given to the development of a comprehensive national program or plan for the prevention and management of suicidal behavior. At the level of the autonomous regions, different plans and strategies have been developed in relation to suicide prevention, but they are heterogeneous in their degree of implementation (20).

The main aim of this study is to determine the relationship between the variation in completed suicide rates in Spain between 2010 and 2022 and active substance use variables. In this study we correlate completed suicide rates and substance use risk factors at a population level without access to individual data of the subjects who committed suicide.

The working hypothesis is that active substance use has a significant influence on the variation in completed suicide rates in Spain between 2010 and 2022. If this hypothesis is verified, the importance of globally reducing the consumption of psychoactive substances as a preventive measure against suicide would be confirmed in the Spanish population.

## Materials and methods

### Data

The incidence rates of suicide deaths for the nineteen Spanish autonomic regions between 2010 and 2022 were obtained from the Spanish Statistical Office (INE) (https://www.ine.es).

The following data were also obtained by means of the INE database for the nineteen Spanish autonomic regions: unemployment rates, masculinity ratio (number of men for every 100 women), average life expectancy, mean age and depression rates (**Table 1**).

**Table 1:** Prevalence of suicide deaths, rates of sociodemographic variables and prevalence of depression obtained from the INE; and rates of variables associated with substance use obtained from the PNSD, in Spain from 2010 to 2022.

	2010	2011	2012	2013	2014	2015	2016	2017	2018	2019	2020	2021	2022
**Suicides**	6.72	6.74	7.49	8.21	8.36	7.73	7.67	7.90	7.57	7.81	8.31	8.45	8.63
**U**	19.86	21.39	24.79	26.09	24.44	22.06	19.63	17.22	15.25	14.10	15.53	14.78	12.92
**MR**	97.61	97.39	97.21	96.92	96.64	96.45	96.33	96.18	96.10	96.08	96.12	96.11	96.10
**MA**	41.09	41.33	41.60	41.87	42.14	42.43	42.67	42.91	43.13	43.34	43.55	43.81	44.07
**ALE**	81.86	82.49	83.21	83.22	82.19	82.37	82.04	82.67	82.53	83.01	82.31	82.43	82.78
**D**					7.36						5.36		
**A-12 months**		72.85		79.40		79.49			70.43		74.15		74.80
**A-30 days**		59.76		65.28		62.75			58.24		57.74		59.46
**A-daily**		8.41		9.01		6.76			6.81		8.94		10.97
**Binge-12 months**		22.14		21.92		16.34			25.79		13.61		16.10
**Binge-30 days**				7.66		5.22			10.08		3.94		5.55
**Audit +**				8.76					6.76		4.51		5.49
**BZD- 12 months**		2.09		14.40		15.76			11.92		12.74		16.93
**BZD- 30 days**		1.26		9.43		9.73			7.89		9.94		12.50
**BZD- daily**				6.11		7.68			6.27		7.68		9.12
**IS- 12 months**		8.65		8.60		8.83			11.12		6.97		9.12
**IS- 30 days**		6.48		5.90		6.46			8.78		4.84		7.45

U, Unemployment; MR, Masculinity ratio; MA, Mean Age; ALE, Average Life Expectancy; D, Depression prevalence; A-12 months: Alcohol use in the last 12 months; A-30 días: Alcohol use in the last 30 days; A-daily: Daily alcohol use in the last 30 days; Binge-12 months: Binge drinking in the last 12 months; Binge -30 days: Binge drinking in the last 30 days; AUDIT+: Positive Alcohol Use Disorder Test (AUDIT) for risky alcohol use; BZD- 12 months: Benzodiacepine use in the last 12 months; BZD-30 days: Benzodiacepine use in the last 30 days; BZD-Daily, Daily benzodiazepine (BZD) use in the last 30 days; IS-12 moths: Illegal substances use in the last 12 months; IS-30 days: Illegal substances use in the last 30 days.

The data on substance use for the same period for the nineteen Spanish autonomic regions come from the survey on alcohol and other drugs in Spain (EDADES) (https://pnsd.sanidad.gob.es/profesionales/sistemasInformacion/home.htm), which the Government Delegation for the National Plan on Drugs has performed biannually since 1995 (**Table 1**). The following variables were included: Alcohol use in the past 12 months, alcohol use in the last 30 days, daily alcohol use in the last 30 days, binge drinking in the last 12 months, binge drinking in the last month, positive Alcohol Use Disorder Test (AUDIT) for risky alcohol use, benzodiazepine (BZD) use in the last 12 months, BZD use in the last month, daily BZD use in the last month, use of illegal substances in the last 12 months and use of illegal substances in the last month. The study focused on variables that measure recent consumption, and not on those that measure lifetime consumption.

### Ethics aspects

The authors complied with all the contents set out in the current legislation on clinical research established in the Declaration of Helsinki, European Convention on Human Rights and Biomedicine and in the UNESCO Universal Declaration on Human Rights. They complied with the requirements established in the Spanish legislation in the field of medical research, the protection of personal data and bioethics and all other requirements set out by Spanish legislation on this topic. The current research contains no human or animal studies.

### Statistical analysis

The temporal trend of completed suicide incidence rates in all the nineteen Spanish autonomic regions was evaluated using a joinpoint regression model. This statistical modelling technique has previously been used successfully in the field of addictions (21, 22). Changes in the time trend are called joinpoints or inflection points. A Poisson distribution model was used in the estimation.

The analysis that has been carried out to obtain the direct relationship between the independent variables and the suicide rate is based on an exploratory analysis, so that the relationship between the independent variables and the suicide rate is graphically described. Using this type of graphs, the pattern or trend of the relationship between the variables has been identified, and non-linear relationships between the variables have also been identified.

Afterwards a more detailed analysis with Generalized Additive Models (GAM) was carried out. GAM is a generalized linear model in which the linear response variable depends linearly on unknown smooth functions of some predictor variables, and interest focuses on inference about these smooth functions. In the application of these models, we obtained that the relationship between the independent variable and the suicide rate varies in a more complex way than a simple linear relationship. The significance implies that there is a statistically significant relationship between the dependent variable and the events but, again, to understand this relationship it is necessary to view the corresponding graph. Therefore, the result of the fitting model is a smoothed curve.

The criterion of statistical significance in all tests was *P ≤* 0.05, established as the maximum acceptable value for the probability of making a type 1 error.

## Results

**Table 1** shows all variables used in the study unified at a national level for clarity reasons.

**Figure 1:**
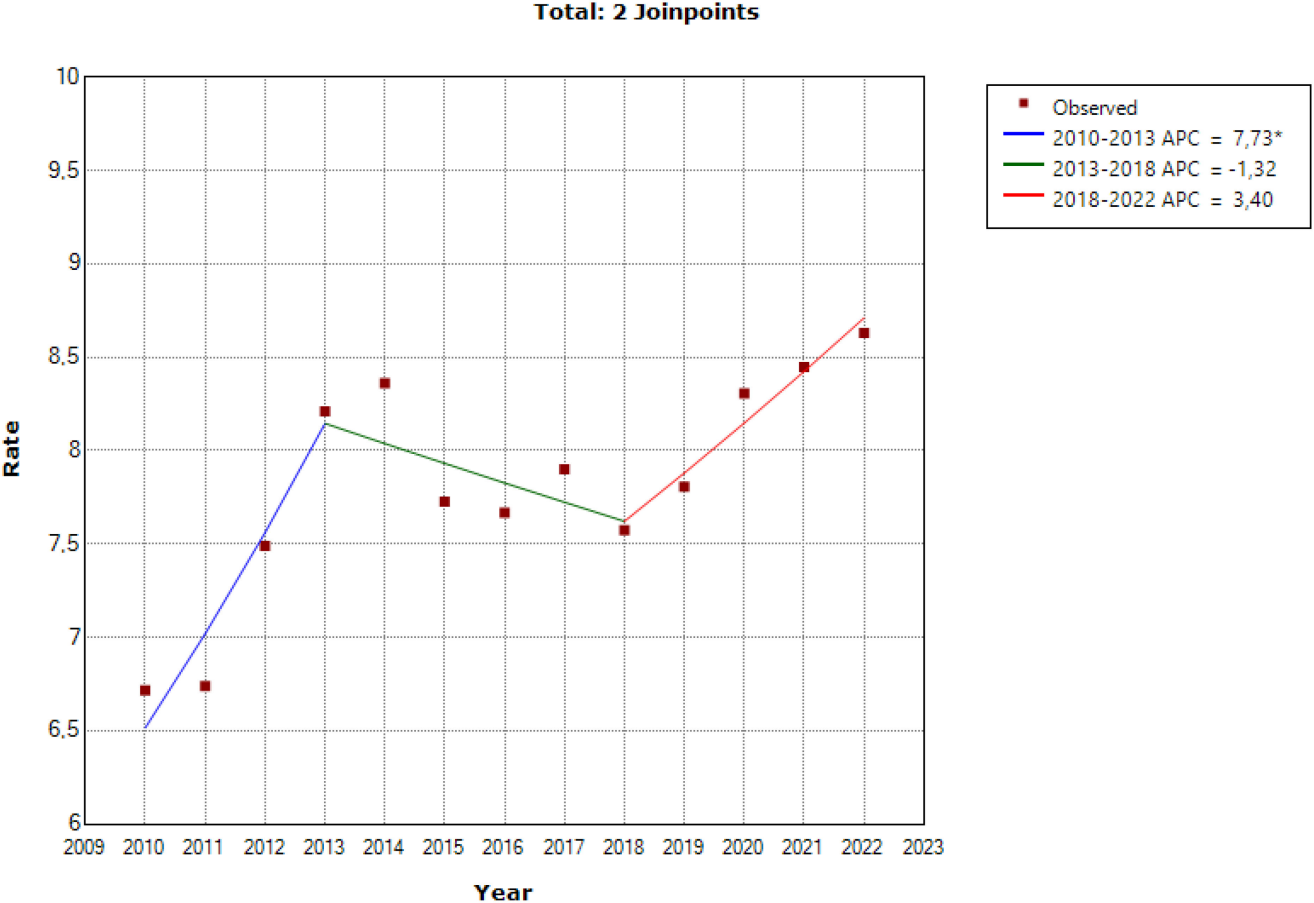
Joinpoint regression model for the completed suicide rates at a unified national level. *Statistical significant.

As can be observed in **Figure 1** the joinpoint regression model showed one significant change point, and another close to significance for the completed suicide rates at a unified national level. These two change points created three different segments: 2010-2013 (APC = 7.7, t = 3.2, p = 0.023), 2013-2018 (APC = -1.3, t = -0.49, p = 0.403), and 2019-2022 (APC = 3.4, t = 2.3, p= 0.07).

**Figure 2:**
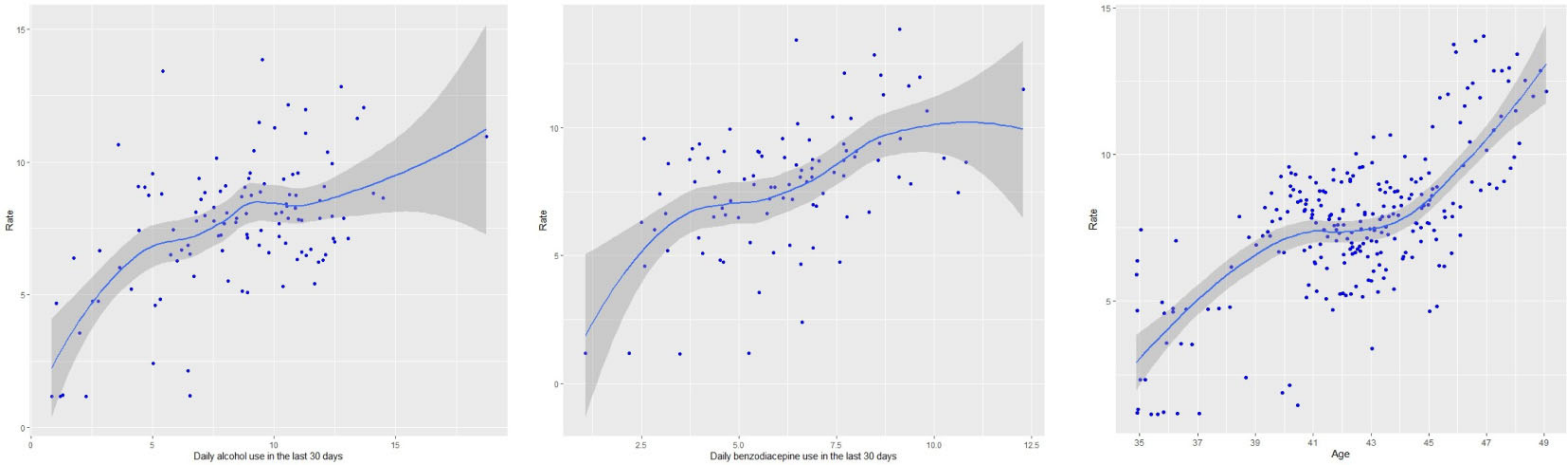
Exploratory analysis graphically for the most relevant independent variables.

**Figure 2** shows the exploratory analysis graphically for the most relevant independent variables: daily alcohol use in the last 30 days, daily BZD use in the last 30 days and age.

**Figure 3:**
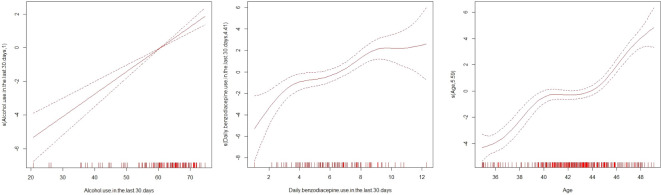
Generalized Additive Models analysis graphically for the most relevant independent variables.

**Figure 3** shows the GAM models for the most relevant independent variables: alcohol use in the last 30 days, daily BZD use in the last 30 days and age. The following independent variables showed that their relationship with the suicide rate varied in a positive significant more complex way than a simple linear relationship: alcohol use in the past 12 months (t = 45.63, p < 0.0001), alcohol use in the last 30 days (t = 39.83, p < 0.0001), daily alcohol use in the last 30 days (t = 39.42, p < 0.0001), binge drinking in the last 12 months (t = 32.79, p < 0.0001), binge drinking in the last month (t = 32.54, p < 0.0001), positive AUDIT for risky alcohol use (t = 27.59, p < 0.0001), BZD use in the last 12 months (t = 37.93, p < 0.0001), BZD use in the last month (t = 38, p < 0.0001), daily BZD use in the last month (t = 35.97, p < 0.0001), use of illegal substances in the last 12 months (t = 33.05, p < 0.0001), use of illegal substances in the last month (t = 32.7, p < 0.0001), mean age (t = 71.66, p < 0.0001) and depression rates (t = 25.94, p < 0.0001).

## Discussion

The results of the evolution of deaths by suicide in Spain reflected in **Figure 1** show the following: the incidence of completed suicides increased progressively between 2010 and 2013. Afterwards, it was stabilized and even decreased slightly until 2018, to increase again since 2018 to 2022. This evolution indicates that the increase in the incidence of deaths due to completed suicide in Spain had begun before the COVID-19 pandemic in 2020. These results are remarkable when interpreting the impact of the pandemic of COVID-19 in completed suicide rates, at least in Spain; especially if we take into account that the results worldwide are not homogeneous, with studies indicating an increase in death rates by suicide during the pandemic (23), while others do not reflect this increase (24).

The results of this study confirm that in Spain, as in other countries (13), there is an important and worrying relationship between alcohol consumption at the population level and the incidence of completed suicide. These previous studies (13) indicated that this relationship occurred in countries where consumption is more oriented towards intoxication and binge drinking and did not occur in countries with a wine culture, that is, daily consumption, not oriented towards drinking large quantities of alcohol in short periods of time with an intoxicating objective. Unfortunately, the data indicate that in Spain the “culture” of alcohol consumption, since the beginning of the century, is transitioning from this last model to a more intoxicating form of consumption (25). Unluckily, this may be leading to an increase in the relationship between recent alcohol consumption and suicide deaths as reflected in our study.

The data from this study indicate that recent alcohol consumption, especially through abuse and binge drinking is associated with a higher incidence of completed suicide. How does this association work? The answer is not simple since there are several mechanisms that explain it: (I) alcohol is a potentially lethal chemical agent in overdose and can be used, alone or in combination, as a suicidal method (13) (II) Furthermore, acute and intense alcohol consumption is a precipitating factor of suicide since it generates impulsivity, emotional instability, irritability, and increases depressive thoughts of hopelessness and suicidal ideation (11, 13). (III) Moreover, chronic abusive consumption of alcohol is a persistent suicidal risk factor since it presents a high comorbidity with other mental disorders, especially depression, which already increases the suicidal risk. Also, this comorbidity increases the suicidal risk beyond the sum of risks each disorder presents separately (9, 14). (IV) Finally, the social, family and work deterioration caused by chronic alcohol abuse increases thoughts of hopelessness and suicidal ideation and increases the risk of the mental disorders such as depression, notably increasing the suicidal risk (9).

In this study, in the same way as for alcohol, recent consumption of BZD is also associated with the incidence of completed suicide in Spain at a population level. Previous international studies have already confirmed this worrying association between BZD consumption and completed suicide (26, 27). A straightforward way of explaining this association is simply pointing out the use of BZDs to treat mental disorders, anxiety and others, which already have an associated suicidal risk, and that therefore this association is the product of a statistical bias (28). However, it must be remembered that BZDs have a potential for abuse and dependence similar to alcohol (29). In this situation of uncontrolled use, BZDs, like alcohol, in their acute excessive consumption in overdose can also be a lethal chemical agent that can be used as a suicide method, and their chronic abuse or dependence is comorbidly associated with other mental disorders with suicidal risk with the paradox that they were initially prescribed to alleviate the symptoms of these disorders. Also, they can also generate a chronic situation of social, family and work deterioration that increases the suicidal risk (27, 28). In summary, BZDs generate a suicidal risk similar to that of alcohol in people with problems of abuse or dependence (29). From this perspective it is easy to understand why the increase in the use and prescription of BZD registered in Spain over recent years is so worrying from the point of view of suicidal risk and completed suicide (25).

**Figures 2**, **3** show how, in the general Spanish population, recent consumption of alcohol and BZD is associated with completed suicide with the same power as age, a well-known and non-modifiable risk factor.

As in previous studies, the consumption of illegal substances is also associated in the current study with an increase in the prevalence of death due to completed suicide at the population level (13, 15). Of all the illegal substances, opioids are the most concerning since they are not only obtained through an illegal route, such as heroin, but also through a medical prescription for pain management, such as fentanyl. Furthermore, in both cases, opioids are very present in the autopsies of people who have committed suicide (15), indicating both their recent daily use and their potential lethality in overdose. Likewise, it must be taken into account that opioid consumption is associated with a chronic scenario of suicidal risk through high comorbidity with psychiatric disorders and social, family and work deterioration. In summary, they generate a similar suicidal risk model such as BZDs and alcohol (13).

In this study, population data on depression, although limited, are also positively associated with the prevalence of deaths due to completed suicide. This relationship is well known in the field of suicide studies (30), and therefore reinforces the external validity of this study.

All these results agree with those obtained by the national EDADES survey carried out by the PNSD. This survey shows how -compared to the general population interviewed- people who indicated problematic cannabis use, or risky alcohol use, or use of opioid analgesics, or use of illegal substances or BZDs had an increased risk of having suicidal ideation, suicide plans or having committed more suicide attempts. If we take into account that all these forms of consumption have increased in Spain during the period of analysis of this study, it is easy to understand the potential relevance of this problem as the prevalence of completed suicide increases. In all likelihood, this entire situation of high population risk can be explained from the perspective of the “Deaths of Despair”, a phenomenon studied especially in the United States, in which the increase in deaths from drug overdose, alcoholic liver disease and suicide is associated with increased stress, physical and emotional pain in people exposed to a rapidly deteriorating social and economic system (31). **Figure 1** of the present study shows an evolution of completed suicide that reinforces this hypothesis since the increases in completed suicide in Spain between 2010 -2013 and from 2018 coincide with periods of socioeconomic worsening. In summary, social and economic deterioration would produce an increase in psychological and physical discomfort that would lead to an increase in the consumption of alcohol, BZD, illegal substances and opioid analgesics. This would seek to attenuate this discomfort, but it would generate a rebound scenario through abuse and addiction that would worsen this physiological and physical discomfort, and that would therefore deteriorate the overall mental state of the population, and would especially increase depression rates, to, finally, generate a state of population hopelessness that triggers suicide.

All these data highlight the need to always take into account the use and abuse of alcohol and other substances when developing a suicidal behavior prevention program if it is to be successful. By assessing suicide risk in alcohol, BZD, illegal substances and opioid analgesics abusers, prevention efforts could be strategically implemented (9, 32).

The limitations of this study are the following: the data on substance use from the PNSD must be improved. It would be necessary to have more variables that address the misuse of BZDs. It would also be important to have variables that address the consumption of the main illegal substances separately. At the same time, it would also be convenient to have more robust depression population data. Finally, the results of this study allow us to establish a worrying relationship between alcohol and substance consumption and completed suicide at a population level, but do not allow us to establish a simple and unidirectional causal relationship at the individual level.

## Conclusions

Reducing the consumption of alcohol, BZD and illegal substances in those people who consume on a more intense and daily basis, especially if they suffer from depression, could help reduce the rates of suicide deaths in Spain. This is because consumption of alcohol, BZD and illegal substances has positively influenced the prevalence of completed suicide in the nineteen Spanish autonomic regions during the period studied.

## Data Availability

Publicly available datasets were analyzed in this study. This data can be found here: www.ine.es; https://pnsd.sanidad.gob.es/profesionales/sistemasInformacion.
